# HLA‐B Leader Dimorphism Predicts BK Polyomavirus Replication After Kidney Transplant

**DOI:** 10.1111/tan.70477

**Published:** 2025-11-20

**Authors:** Aurélien Aubry, Baptiste Demey, François Helle, Ophélie Fourdinier, Julien Lion, Judith Desoutter, Etienne Brochot, Nicolas Guillaume

**Affiliations:** ^1^ Department of Virology Amiens University Hospital Amiens France; ^2^ UR 4294, Agents Infectieux, Résistance et Chimiothérapie (AGIR) Jules Verne University of Picardie Amiens France; ^3^ Department of Nephrology Dialysis Transplantation Amiens University Hospital Amiens France; ^4^ Department of Histocompatibility Amiens University Hospital Amiens France; ^5^ EA4666 HEMATIM Jules Verne University of Picardie Amiens France

**Keywords:** HLA‐B antigens, kidney transplantation, polyomavirus BK, posttransplant infections

## Abstract

BK polyomavirus (BKPyV) and Cytomegalovirus (CMV) often replicate after kidney transplantation, with management limited to reducing immunosuppression and risking rejection. Among genetic factors that may modulate the antiviral response, HLA evolutionary divergence (HED, allowing a greater diversity of antigen presentation) and the HLA‐B leader dimorphism (MM, MT, TT, influencing NK cell–mediated immunity) are interesting. We aimed to compare these parameters between patients who experienced viral replication within the first post‐transplant year and those who did not. We analysed 306 kidney transplants performed between 2019 and 2023 at Amiens University Hospital. HLA typing was performed in recipients and donors to determine HED (loci A, B, C, DRB1, DQB1) and HLA‐B leader dimorphism. 22.5% of the recipients (69/306) developed BKPyV possible nephropathy (defined by urinary PCR > 7 log_10_ copies/mL or DNAemia) and 40.2% (123/306) developed CMV infection during the first year. In a multivariate analysis, a donor–recipient HLA‐B leader mismatch was protective against BKPyV replication (OR = 0.48; *p* = 0.02), whereas no similar association was observed for CMV replication (OR = 1.42; *p* = 0.169). HLA‐B leader dimorphism could serve as a predictor of BKPyV replication risk, if confirmed in independent cohorts.

## 1 Introduction

In the context of kidney transplantation, the use of immunosuppressive treatments preventing graft rejection exposes patients to a risk of opportunistic infections [1, 2], such as Cytomegalovirus (CMV) and BK Polyomavirus (BKPyV) replication. These viral reactivations are major causes of morbidity and graft dysfunction in Kidney Transplant Recipients (KTR), reflecting the delicate balance between viral control and immunosuppression. BKPyV can cause BKPyV associated nephropathy (BKPyVAN) in 1%–10% of the KTR and guidelines classify patients into possible, probable, presumptive and proven BKPyVAN [3]. On the other hand, CMV infection is defined by viral replication, whereas organ damage characterises CMV disease [4]. The risk factors for these viral replications are still not fully understood. Because these viruses are controlled primarily by the host immune system, immunity plays a central role in determining post‐transplant outcomes. For CMV, pre‐transplant humoral immunity is well established as protective, with seropositivity in the recipient conferring a low risk of replication [5]. More recently, the importance of cellular immunity has been highlighted, particularly through ELISpot tests, which can predict patients at high risk due to an insufficient immune response [6, 7]. For BKPyV, both humoral and cellular immunity have demonstrated their value. Humoral immunity prior to transplantation is predictive of protection against BKPyV replication [8, 9], whereas cellular immunity plays a key role in viral clearance [10].

Given this central role of immune function, the study of HLA, which mediate antigen presentation to immune cells, has proven particularly relevant in virology [11, 12]. In transplantation, certain recipient HLA serotypes may confer protection against viral reactivation, like HLA‐B51 for BKPyV [13] (13), and HLA‐A01, A02 for CMV [14], whereas others appear linked to an increased risk (HLA‐B35 Cw4 for BKPyV [15]). In addition, recent work in healthy individuals has suggested that HLA‐B leader genotypes ‐TT or ‐MT are associated with a more efficient anti‐BKPyV immune response [16]. However, kidney transplantation represents a particular immunological context with donor and recipient, and it remains unknown whether HLA‐B leader genotype affects viral replication in this setting.

Considering donor and recipient HLA genes in the context of kidney transplantation, a recent study provides evidence for the functional relevance of donor HLA‐DQ diversity, assessed by HLA evolutionary divergence (HED), in the control of BKPyV infection [17]. Indeed, HED quantifies the sequence divergence between the peptide binding domains of the two alleles of a given locus: a higher HED indicates a broader immunopeptidome and potentially a more robust antiviral response. This finding highlights that donor HLA characteristics may also contribute to antiviral control, underscoring the importance of considering both donor and recipient HLA profiles in transplantation studies. Supporting this concept, a previous study showed that compatibility between donor and recipient for HLA‐A02, B44, and DR15 reduces the risk of BKPyV replication [18].

Considering these data, we hypothesised that genetic determinants of immune diversity—specifically HED and HLA‐B leader status—could modulate the risk of opportunistic viral replication after kidney transplantation. Since HLA typing is routinely performed prior to transplantation, these data could provide a simple and effective tool for assessing the risk of viral replication post‐transplantation. The objective of this study is therefore to analyse, within a cohort of donor‐recipient pairs, the association between certain genetic parameters (HED, HLA‐B leader status) and the occurrence of BKPyV and CMV replication after kidney transplantation.

## 2 Methods and Materials

### 2.1 Cohort

This study is based on the analysis of a retrospective cohort of kidney transplant recipients. The inclusion criterion was kidney transplantation performed between January 2019 and December 2023 at Amiens‐Picardie University Hospital. Exclusion criteria included graft removal, loss to follow‐up or death occurring within the first post‐transplant year, absence of post‐transplant virological monitoring (CMV and BKPyV PCR during follow‐up), and finally, patient refusal to consent to the use of its personal data. This non‐interventional study used data collected strictly within routine clinical care and was approved by Amiens University Hospital Research and Innovation Department (DRCI), under registration number PI2022‐843‐0079.

Patients were classified based on prospective CMV and BKPyV PCR monitoring performed as part of routine clinical care, with retrospective data collection. CMV infection is defined as the detection of CMV nucleic acid in any body fluid [4]. Therefore, patients with at least one quantifiable CMV PCR (either plasma or whole blood) were classified as CMV positive. Possible BKPyV nephropathy is defined by a high‐level BKPyV viral load in urine, specifically BKPyV‐DNAuria greater than 7 log_10_ copies/mL, in the absence of detectable plasma BKPyV DNAemia. Patients were considered BKPyV positive if they had either at least one positive plasma PCR or a urine viral load exceeding 7 log_10_ copies/mL [3].

### 2.2 PCR Assays

BKPyV PCR was performed on urine or plasma samples using the AltoStar BKV PCR Kit (Altona Diagnostics, Hamburg, Germany, quantification threshold = 200 copies/mL). CMV PCR assays changed during the study period: Until July 2021, CMV PCR was performed on whole blood using the Abbott RealTime CMV assay (Abbott Park, Illinois, USA, quantification threshold = 40 copies/mL). From July 2021, it was performed on plasma using the Cobas CMV assay (Roche Molecular Systems, Pleasanton, CA, USA, quantification threshold = 34.5 UI/mL). Since the definition of CMV infection does not distinguish between plasma and whole blood PCR results, CMV infection cases were defined in this study by a quantifiable CMV PCR in either plasma or whole blood. This approach was supported by method validation and consistent with the literature [19], despite differences in quantification between the two assays. Therefore, no quantitative viral load analysis was performed in this cohort; patients were classified based on positive or negative CMV PCR results in plasma or whole blood.

### 2.3 HLA Typing and Analysis

High‐resolution HLA typing was available for recipients and performed by next‐generation sequencing (NGS) provided by Werfen (Mia Fora NGS Mflex HLA, Norcross, Georgia). For donors, HLA typing was performed at low resolution using quantitative PCR (qPCR) provided by CareDX (Olerup QTYPE 11, West Chester, PA), and alleles were imputed using the HaploSFHI online tool (www.sfhitools.fr/HaploSFHI) as reported [20].

The HLA Evolutionary Divergence (HED) score was calculated using the tool developed by Lima et al. [21], accessible via the web application (https://txor.shinyapps.io/ched/). The HED score quantifies the sequence divergence between alleles at the same HLA locus, computing HED scores for the five classical HLA genes (HLA‐A, B, C, DRB1 and DQB1). In addition, HLA‐B leader mismatches were evaluated using the HLA‐B Leader Mismatch Calculator (IPD‐IMGT/HLA database: https://www.ebi.ac.uk/ipd/imgt/hla/matching/b_leader/).

### 2.4 Data Collected for Statistical Analysis

Global and locus‐specific HED scores (A, B, C, DRB1 and DQB1), were used for statistical analysis, as donor and recipient HLA‐DQA1 profiles (heterozygous DQα01/non‐DQα01 vs. others), HLA‐B leader status and presence or absence of HLA‐B leader match between donor and recipient. Other clinical data were collected for multivariate analysis, as pre‐transplant general data (donor and recipient sex and age, recipient body mass index (BMI), smoking status, residual urine output before transplant, pre‐transplant CMV and EBV serostatus, and presence of donor‐specific antibodies (DSA) at day 0) and transplant‐related data (cold ischemia time, postoperative creatinine nadir, induction therapy—basiliximab or ATG, maintenance immunosuppression—tacrolimus or cyclosporine, corticosteroid use during maintenance, and donor type—living or deceased).

### 2.5 Statistical Analysis

Univariate statistical analyses were performed using R software (version 4.3.2). For each binary variable (CMV or BKPyV status), all other variables were tested individually for association. For continuous dependent variables, normality was assessed using the Shapiro‐Wilk test within each group. If both groups showed normally distributed data, Student's *t*‐test was used. Otherwise, the Wilcoxon rank‐sum test was applied. For categorical variables, the chi‐squared test was used when all expected counts were ≥ 5; otherwise, Fisher's exact test with simulated *p*‐values was performed. Multivariate analysis was performed using logistic regression.

For multivariable analyses, we included variables with a univariate *p*‐value < 0.05, whereas respecting the conventional rule of approximately one covariate per 10 events (i.e., up to 7 variables for BKPyV [69 events] and up to 12 for CMV [123 events]). This resulted in eight variables for CMV. For BKPyV, only three variables met the *p* < 0.05 threshold; to reduce the risk of missing potential confounders, we therefore expanded the selection to the seven variables with the lowest univariate *p*‐values. Absence of collinearity between variables was verified.

## 3 Results

### 3.1 Inclusion

**FIGURE 1:**
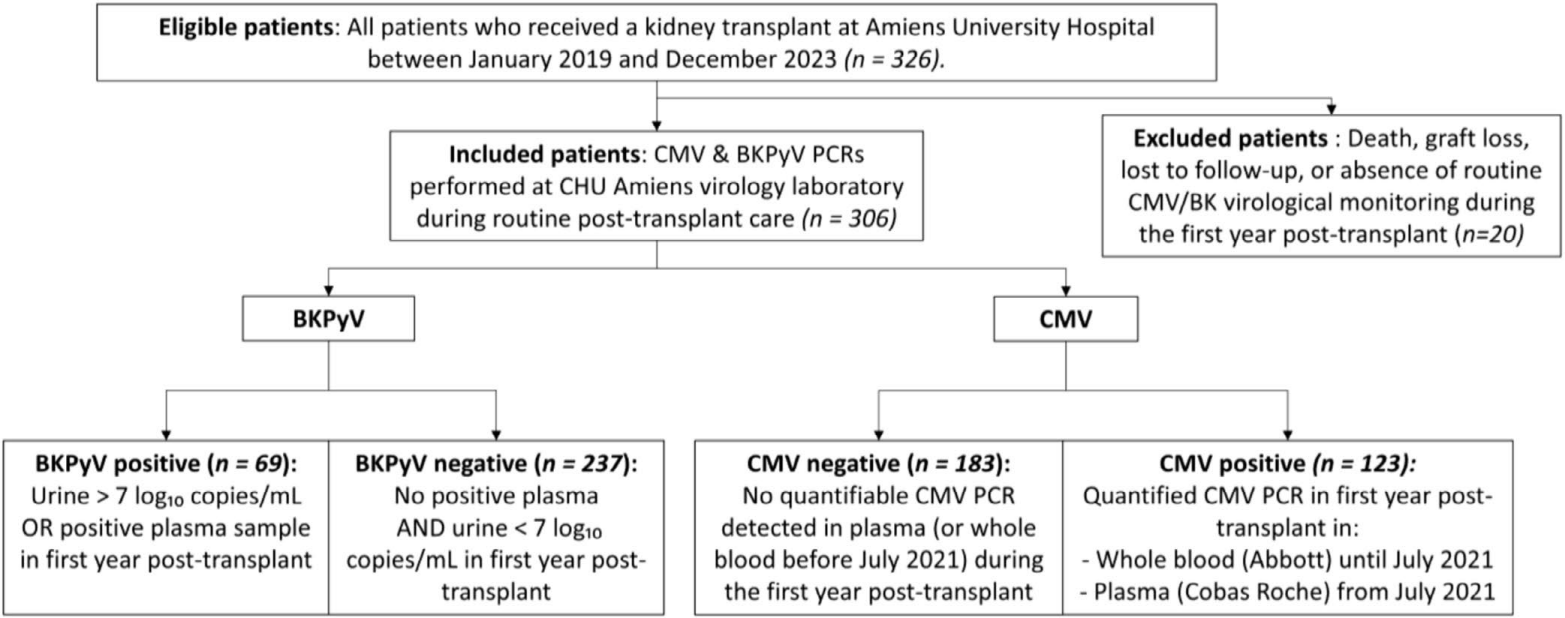
Flowchart illustrating the inclusion of patients in the study cohort.

A total of 326 KTR were eligible for inclusion in this study, of whom 306 were included after applying exclusion criteria (Figure 1). Among them, the prevalence of possible BKPyV nephropathy (BKPyV PCR in urine > 7 log_10_ copies/mL or BKPyV DNAemia) was 22.5% (69/306), and the prevalence of CMV infection was 40.1% (123/306). Replication of both BKPyV and CMV during the first post‐transplant year was not uncommon (22 patients—7.2% of the cohort) but did not occur more frequently than expected by chance (Chi‐squared test, *p* = 0.2343). Having a CMV infection did not alter the risk of BKPyV infection, and vice versa.

For the univariate analysis (Supplementary Data 1) the parameters that differ significantly between BKPyV‐positive and BKPyV‐negative patients in terms of HED and HLA‐B leader status were: recipient HED‐B, donor‐recipient HLA‐B leader mismatch and donor HED‐A. A multivariate analysis was then performed including the seven parameters with the smallest *p*‐values after verifying the absence of collinearity. The only parameter that remained significant in the multivariate model was the donor‐recipient HLA‐B leader mismatch, which appeared protective (Odd Ratio = OR = 0.48, *p* = 0.017, Table 1), indicating that patients with an HLA‐B leader mismatch had approximately half the risk of BKPyV replication. The most protective configuration was observed in TT donor/MT recipient pairs (BKPyV in 5/56 cases, 8.9%), compared with TT donor/TT recipient pairs (BKPyV in 31/94 cases, 33%).

**TABLE 1:** Multivariate analysis results for factors associated with possible BKPyV nephropathy (logistic regression model).

Variable	*p*	OR	OR 2.5	OR 97.5
HLA‐B leader status: mismatch donor/recipient	0.017	0.482	0.262	0.871
HED A donor	0.072	0.93	0.86	1.006
Post‐transplant creatinine nadir (μmol/L)	0.168	1.001	1	1.002
HED B recipient	0.214	0.936	0.843	1.039
HED B donor	0.267	1.052	0.963	1.154
HLA‐B leader status (recipient)—MT	0.431	0.64	0.214	2.01
HLA‐B leader status (recipient)—TT	0.81	0.885	0.336	2.502

In terms of HED and HLA‐B leader status, only one factor showed a moderate association with CMV in the univariate analysis: HLA‐B leader mismatch, this time with a rather deleterious effect (*p* = 0.045). However, it did not remain significant in the multivariate analysis, whereas several significant differences were still observed for clinical parameters (Table 2 and Supplementary Data 2): recipient CMV serostatus (OR = 2.15; *p* = 0.003), male recipient sex (OR = 0.54; *p* = 0.021), and presence of donor‐specific antibodies (DSA) at day 0 (OR = 2.14; *p* = 0.05).

**TABLE 2:** Multivariate analysis of risk factors for CMV infection within the first year post‐transplantation.

Variable	*p*	OR	OR 2.5	OR 97.5
CMV serostatus (recipient)	0.003	2.151	1.296	3.602
Sex (recipient‐Male)	0.021	0.542	0.320	0.911
DSA at day 0	0.050	2.141	1.007	4.654
Post‐transplant creatinine nadir (μmol/L)	0.107	1.001	1.000	1.002
Cold ischemia time (min)	0.137	1.001	1.000	1.001
HLA‐B leader status: mismatch donor/recipient	0.169	1.421	0.862	2.347
Age (donor)	0.330	1.015	0.985	1.048
Age (recipient)	0.375	1.013	0.985	1.042

## 4 Discussion

BKPyV replication is a major complication in kidney transplantation, as its treatment relies on reducing immunosuppressants, which increases the risk of rejection, whereas lack of treatment exposes patients to irreversible BKPyVAN. In this context, preventing replication is essential but challenging due to the high seroprevalence of BKPyV in donors and recipients and imperfect knowledge of risk factors. This study aims to evaluate the relevance of HLA evolutionary divergence (HED) and HLA‐B leader dimorphism in the risk of BKPyV replication and CMV infection, another significant complication following transplantation.

In this cohort of 306 kidney transplant recipients (KTRs) at Amiens‐Picardie University Hospital, virological follow‐up during the first post‐transplant year revealed a prevalence of 22.5% for possible BKPyV nephropathy (69/306) and 40.2% for CMV infection (123/306), broadly consistent with local epidemiology. Indeed, BKPyV prevalence was previously estimated at 19% (47/248) in our centre between 2013 and 2016 (using slightly different criteria: urinary PCR > 6.7 log_10_ copies/mL) [22], and CMV infection prevalence was 36.6% in 2008 in another French cohort [23].

The most striking finding of this study is the potential role of HLA‐B leader dimorphism in predicting the risk of BKPyV replication. In accordance with a previous report implicating HLA‐B51 in protection against BKPyV replication [13], our finding that higher recipient HED‐B values correlate with lower BKPyV incidence (median HED‐B: 8.31 in recipients without BKPyV versus 7.47 in those with BKPyV; Wilcoxon rank‐sum test, *p* = 0.007) further supports the importance of HLA‐B in anti‐BKPyV immunity. However, this association did not remain statistically significant after adjustment in multivariable analysis. Notably, a donor–recipient mismatch at the HLA‐B leader position was associated with a substantially reduced risk of possible BKPyVAN (OR = 0.49), corresponding to an approximate 50% risk reduction.

One of the interesting findings is the protective effect of HLA‐B leader mismatch between donor and recipient. This is particularly noteworthy because the HLA‐B leader has already been shown to influence anti‐BKPyV immunity in healthy individuals [16]. Interestingly, it was expected that the presence of a ‐T allele would be protective, which is not clearly observed here. In our cohort, the MT genotype is moderately protective in KTR (40.93% of KTR without BKPyV versus 27.54% of KTR with BKPyV, Fisher's exact test, *p* = 0.048), whereas the TT genotype is more common in patients with BKPyV. Indeed, the TT genotype could be deleterious with regard to BKPyV, given that it accounts for 60.87% of patients with BKPyV in this cohort, compared to 49.37% of patients without BKPyV and 51.6% in the general population, according to an American study [24]. However, the complexity of transplantation lies in the interaction between donor and recipient, and in this cohort, the recipient‐donor mismatch appears to be the most significant factor. This raises questions about the underlying mechanisms.

HLA class I molecules, including HLA‐B, function as immune checkpoints for NK cells, and HLA‐B leader mismatch could therefore influence NK cell activity, based on existing literature. Indeed, the HLA‐B leader peptide dimorphism modulates HLA‐E expression: ‐21M carriers exhibit high HLA‐E expression, promoting NK cell education via the inhibitory receptor CD94:NKG2A, whereas ‐21T carriers show reduced HLA‐E expression, favouring NK cell education through Killer‐cell Immunoglobulin‐like Receptors (KIRs), which may be more effective in certain viral contexts [25]. HLA‐E polymorphism has already been described as important for BKPyV [26], suggesting that its stability via HLA‐B leader could also be relevant. Furthermore, it has been shown that greater mismatches in leader peptides between donor and recipient in kidney transplantation are associated with higher HLA‐E expression, which correlates with infiltration of NK cells and CD8+ T cells [27] that may contribute to an improved anti‐BKPyV response. Indeed, NK cells appear to be relevant for anti‐BKPyV immunity, as HLA‐B35‐Cw4, an inhibitory ligand for NK cells, has been associated with increased BKPyV DNAemia [15], and by evidence that the presence of specific activating KIRs confers protection [28]. The role of CD8+ T cells in anti‐BKPyV immunity is well established too [29]. The exact mechanism by which HLA‐B leader mismatch enhances anti‐BKPyV immunity remains to be elucidated, and it is possible that it may also be linked to a higher risk of overall alloreactivity, potentially increasing the risk of graft rejection. Indeed, in the setting of haematopoietic stem cell transplantation, such mismatches have been associated with an increased risk of acute graft‐versus‐host disease [30]. This finding suggests that HLA‐B leader mismatching may enhance overall immune activation, which could be advantageous for antiviral control but raises important questions regarding graft tolerance.

Finally, the relevance of HED in controlling BKPyV is reinforced by a recent study showing that greater HLA‐DQ, notably HLA‐DQA1 genes, divergence in the donor correlates with a broader BKPyV immunopeptidome and a reduced risk of BKPyV replication [17]. For these authors, having a heterozygous DQα01/non‐DQα01 donor strongly protected against the risk of post‐transplant BKPyV DNAemia compared to other combinations. In our cohort, we applied the same approach but we did not find an association between heterozygous DQα01/non‐DQα01 donor and BKPyV replication (Chi‐square test, *p* = 0.667). These data confirm the value of continuing investigations, combining HED and HLA‐B leader dimorphism analyses in larger multicenter cohorts, in order to consolidate the evidence and clarify the underlying immunological mechanisms.

It is also noteworthy that neither HLA‐B leader dimorphism nor HED appeared to be associated with CMV infection in this cohort. The only variables linked to CMV infection risk were predominantly clinical factors. In established literature, donor/recipient serostatus (D+/R−), type of induction therapy (notably ATG), recipient age, lymphopenia and exposure to mycophenolate are all well‐recognised risk factors for CMV infection [31]. In our study, CMV‐seronegative status of the recipient prior to transplantation was confirmed as a significant risk factor [32, 33]. Female sex, previously associated with greater tissue involvement [34], and the presence of donor‐specific antibodies at day 0 (DSA; not widely reported in the literature and with borderline significance, *p* = 0.05) were also observed.

To our knowledge, this is the first study to investigate the association between HLA‐B leader dimorphism and the risk of BKPyV replication after kidney transplantation. These findings reinforce the potential relevance of HLA‐B leader genotyping, recently highlighted in healthy donors [16]. However, several limitations must be acknowledged. First, the retrospective nature of the study implies reliance on available routine follow‐up data, which were sometimes incomplete and could have led to false negatives in cases of suboptimal virological monitoring. Clinical parameters were included in the analysis to minimise confounding factors as much as possible through multivariate modelling; however, the analysis cannot be entirely exhaustive, and other potential confounders may exist, particularly immunosuppressive (IS) drug levels throughout the first year post‐transplantation. Second, the HaploSFHI algorithm imputes second‐field HLA typings from low‐ or intermediate‐resolution ones, with an expected risk of error [20]. According to the authors, percentages of correctly imputed typings were 93.82% for HLA‐A, 91.68% for HLA‐B, 96.38% for HLA‐C, 82% for HLA‐DRB1 and 94.76% for HLA‐DQB1. HaploSFHI outperformed reference tools Haplostats, HMA‐EMMA and HLA‐Upgrade across all loci. All tools performed suboptimally for DRB1; the explanation relies mainly on a few serological‐level typings for which the second field is difficult to predict, due to the presence of several frequent DRB1 alleles that are hardly distinguishable even when using linkage disequilibrium. Nevertheless, incorrect second‐field typings did not appear to affect eplet repertoires, limiting their impact on HED analysis and HLA‐B leader classification. Finally, as this was a single‐centre study, external validation in an independent, ideally prospective cohort is warranted.

Pre‐transplant HLA data could help identify patients at increased risk and justify intensified post‐transplant monitoring. Furthermore, combined genetic analyses of the HLA‐B leader and its interacting partners (KIR, HLA‐E, etc.) may prove valuable, given the apparent influence of these factors on anti‐BKPyV immunity, although the underlying mechanisms remain to be elucidated.

## 5 Conclusion

In conclusion, our results highlight the potential value of incorporating HLA‐B leader dimorphism into pre‐transplant risk stratification, which could help to adapt virological surveillance and preventive strategies for BKPyV in KTR.

## Author Contributions

A.A. performed statistical analyses, drafted the manuscript, and coordinated the writing process. E.B. and N.G. conceived the study. Data extraction from clinical records were performed by E.B., N.G., B.D., J.D. and J.L., and all co‐authors critically revised the manuscript and approved the final version.

## Ethics Statement

This non‐interventional study utilised data collected during routine clinical care and was approved by the Amiens University Hospital Research and Innovation Department (DRCI, registration number PI2022‐843‐0079) in compliance with the French MR‐004 framework, ensuring the protection of patient information and data confidentiality.

## Conflicts of Interest

The authors declare no conflicts of interest.

## Supporting information


**Data S1:** tan70477‐sup‐0001‐supinfo.docx.

## Data Availability

The data that support the findings of this study are derived from patient medical records and are subject to ethical and legal restrictions. Therefore, the raw data are not publicly available.
