# Supplementary material for: HLA‐B Leader Dimorphism Predicts BK Polyomavirus Replication After Kidney Transplant
Source: HLA. 2025 Nov 20;106(5):e70477. doi: 10.1111/tan.70477 (PMC12634932; doi:10.1111/tan.70477)
Supplement: Supplementary file 1 — Data S1: tan70477‐sup‐0001‐supinfo.docx. [file TAN-106-e70477-s001.docx]

**Supplementary data**

*Supplementary Data 1: Table of univariate statistical analyses with the presence of possible BKPyV nephropathy during the first post-transplant year as the outcome variable. Quantitative data are presented as mean (SD); qualitative data as n (%).*

|  | | BKPyV Negative (criteria not met) | BKPyV positive (Urine >7 log10/mL and/or Plasma positive during follow-up) | *p-value* |
| --- | --- | --- | --- | --- |
| **HLA** | | | | |
| HED_A-Recipient | | 6.82 (3.52) | 6.83 (3.43) | 0.762^1^ |
| HED_B-Recipient | | 8.14 (3.20) | 6.93 (3.45) | **0.007^1^** |
| HED_C-Recipient | | 4.52 (2.22) | 4.47 (2.20) | 0.822^1^ |
| HED_DRB1-Recipient | | 9.85 (5.33) | 9.33 (5.14) | 0.435^1^ |
| HED_DQB1-Recipient | | 10.30 (6.42) | 9.51 (6.75) | 0.419^1^ |
| HED_total-Recipient | | 39.49 (12.50) | 36.97 (12.25) | 0.111^1^ |
| Heterozygous DQα01/non-DQα01 (recipient) | No | 119 (50.21%) | 42 (60.87%) | 0.184^2^ |
|  | Yes | 115 (48.52%) | 27 (39.13%) |  |
|  | Na | 3 (1.27%) | 0 (0%) |  |
| HED_A-Donor | | 6.67 (3.48) | 5.61 (3.50) | **0.033^1^** |
| HED_B-Donor | | 8.10 (3.32) | 8.61 (3.25) | 0.132^1^ |
| HED_C-Donor | | 4.76 (2.26) | 4.51 (2.23) | 0.312^1^ |
| HED_DRB1-Donor | | 10.07 (5.02) | 9.73 (5.34) | 0.574^1^ |
| HED_DQB1-Donor | | 10.25 (6.17) | 9.75 (6.69) | 0.643^1^ |
| HED_total-Donor | | 39.84 (11.34) | 38.20 (10.39) | 0.215^1^ |
| Heterozygous DQα01/non-DQα01 (donor) | No | 127 (53.59%) | 39 (56.52%) | 0.667^2^ |
|  | Yes | 110 (46.41%) | 30 (43.48%) |  |
| HLA-B leader status (recipient) | MM | 20 (8.44%) | 8 (11.59%) | 0.109^2^ |
|  | MT | 97 (40.93%) | 19 (27.54%) |  |
|  | TT | 117 (49.37%) | 42 (60.87%) |  |
|  | NA | 3 (1.27%) | 0 (0%) |  |
| HLA-B leader status (donor) | MM | 24 (10.13%) | 7 (10.14%) | 0.731^2^ |
|  | MT | 91 (38.40%) | 23 (33.33%) |  |
|  | TT | 122 (51.48%) | 39 (56.52%) |  |
| HLA-B leader status: match donor/recipient | Match | 104 (43.88%) | 43 (62.32%) | **0.010^2^** |
|  | Mismatch | 133 (56.12%) | 26 (37.68%) |  |
| **Recipient** | | | | |
| Age (Years) |  | 54.91 (14.22) | 53.82 (12.59) | 0.457^1^ |
| Sex | Female | 92 (38.82%) | 23 (33.33%) | 0.492^2^ |
|  | Male | 145 (61.18%) | 46 (66.67%) |  |
| Weight (kg) |  | 75.85 (14.42) | 77.69 (16.15) | 0.396^3^ |
| BMI |  | 26.08 (4.12) | 26.54 (4.41) | 0.441^3^ |
| Smoking status | Never | 122 (51.48%) | 33 (47.83%) | 0.758^2^ |
|  | Former | 64 (27%) | 20 (28.99%) |  |
|  | Current | 43 (18.14%) | 15 (21.74%) |  |
|  | NA | 8 (3.38%) | 1 (1.45%) |  |
| CMV serostatus | Negative | 107 (45.15%) | 30 (43.48%) | 0.914^2^ |
|  | Positive | 130 (54.85%) | 39 (56.52%) |  |
| EBV serostatus | Negative | 11 (4.64%) | 2 (2.90%) | 0.740^4^ |
|  | Positive | 226 (95.36%) | 67 (97.10%) |  |
| Residual diuresis (ml/24h) | <500mL | 89 (37.55%) | 23 (33.33%) |  |
|  | >500mL | 133 (56.12%) | 35 (50.72%) |  |
|  | NA | 15 (6.33%) | 11 (15.94%) |  |
| DSA at day 0 | No | 206 (86.92%) | 63 (91.30%) | 0.439^2^ |
|  | Yes | 31 (13.08%) | 6 (8.70%) |  |
| **Donor** | | | | |
| Sex | Female | 93 (39.24%) | 30 (43.48%) | 0.595^2^ |
|  | Male | 142 (59.92%) | 38 (55.07%) |  |
|  | NA | 2 (0.84%) | 1 (1.45%) |  |
| Age (years) |  | 54.88 (13.02) | 54.46 (12.38) | 0.745^1^ |
| Living donor | No | 218 (91.98%) | 64 (92.75%) | 1.000^2^ |
|  | Yes | 19 (8.02%) | 5 (7.25%) |  |
| **Transplantation** | | | | |
| Cold ischemia time (min) |  | 689.93 (334.39) | 702.94 (357.24) | 0.865^1^ |
| ATG induction | No | 177 (74.68%) | 52 (75.36%) | 1.000^2^ |
|  | Yes | 60 (25.32%) | 17 (24.64%) |  |
| Post-transplant creatinine nadir (µmol/L) |  | 236.69 (178) | 280.14 (211.45) | 0.175^1^ |
| Tacrolimus / Cyclosporine | None | 1 (0.42%) | 0 (0%) | 0.530^4^ |
|  | Ciclosporine | 13 (5.49%) | 6 (8.70%) |  |
|  | Tacrolimus | 223 (94.09%) | 63 (91.30%) |  |
| Maintenance with MMF | No | 7 (2.95%) | 1 (1.45%) | 0.688^4^ |
|  | Yes | 230 (97.05%) | 68 (98.55%) |  |
| Maintenance corticosteroids | No | 139 (58.65%) | 44 (63.77%) | 0.533^2^ |
|  | Yes | 98 (41.35%) | 25 (36.23%) |  |
| Quantitative data as mean (SD); qualitative data as n (%)  ¹ Wilcoxon rank-sum test; ² Chi-squared test; ³ Student’s t-test; ⁴ Fisher’s exact test | | | | |

*Supplementary Data 2: Table of univariate statistical analyses with the presence CMV infection during the first post-transplant year as the outcome variable. Quantitative data are presented as mean (SD); qualitative data as n (%).*

|  | | CMV negative | CMV positive | *p-value* |
| --- | --- | --- | --- | --- |
| **HLA** | | | | |
| HED A-Recipient | | 6.93 (3.57) | 6.68 (3.38) | 0.3533^1^ |
| HED B-Recipient | | 7.75 (3.46) | 8.04 (3.04) | 0.4956^1^ |
| HED C-Recipient | | 4.32 (2.24) | 4.78 (2.15) | 0.0579^1^ |
| HED DRB1-Recipient | | 9.31 (5.44) | 10.37 (5) | 0.1602^1^ |
| HED DQB1-Recipient | | 9.97 (6.54) | 10.34 (6.45) | 0.5757^1^ |
| HED total-Recipient | | 38.06 (13.20) | 40.21 (11.23) | 0.2521^1^ |
| Heterozygous DQα01/non-DQα01 (recipient) | No | 101 (55.19%) | 60 (48.78%) | 0.2549^2^ |
|  | Yes | 79 (43.17%) | 63 (51.22%) |  |
|  | Na | 3 (1.64%) | 0 (0%) |  |
| HED A-Donor | | 6.30 (3.46) | 6.62 (3.58) | 0.2740^1^ |
| HED B-Donor | | 8.27 (3.39) | 8.14 (3.19) | 0.5469^1^ |
| HED C-Donor | | 4.90 (2.12) | 4.42 (2.41) | 0.1481^1^ |
| HED DRB1-Donor | | 9.84 (5.24) | 10.21 (4.85) | 0.6852^1^ |
| HED DQB1-Donor | | 10.39 (6.32) | 9.75 (6.23) | 0.2896^1^ |
| HED total-Donor | | 39.70 (11.47) | 39.14 (10.66) | 0.5062^1^ |
| Heterozygous DQα01/non-DQα01 (recipient) (donor) | No | 100 (54.64%) | 66 (53.66%) | 0.9579^2^ |
|  | Yes | 83 (45.36%) | 57 (46.34%) |  |
| HLA-B leader status (recipient) | MM | 17 (9.29%) | 11 (8.94%) | 0.9929^2^ |
|  | MT | 69 (37.70%) | 47 (38.21%) |  |
|  | TT | 95 (51.91%) | 64 (52.03%) |  |
|  | na | 2 (1.09%) | 1 (0.81%) |  |
| HLA-B leader status (donor) | MM | 21 (11.48%) | 10 (8.13%) | 0.2195^2^ |
|  | MT | 73 (39.89%) | 41 (33.33%) |  |
|  | TT | 89 (48.63%) | 72 (58.54%) |  |
| HLA-B leader status : match donor/recipient | Match | 97 (53.01%) | 50 (40.65%) | **0.0450^2^** |
|  | Mismatch | 86 (46.99%) | 73 (59.35%) |  |
| Recipient | | | | |
| Age (Years) | | 52.86 (13.59) | 57.34 (13.88) | **0.0064^1^** |
| Sex | Female | 60 (32.79%) | 55 (44.72%) | **0.0464^2^** |
|  | Male | 123 (67.21%) | 68 (55.28%) |  |
| Weight (kg) | | 76.87 (14.37) | 75.37 (15.47) | 0.3929^3^ |
| BMI | | 26.26 (4.08) | 26.08 (4.36) | 0.7180^3^ |
| Smoking status | Never | 87 (47.54%) | 68 (55.28%) | 0.0959^2^ |
|  | Former | 51 (27.87%) | 33 (26.83%) |  |
|  | Current | 42 (22.95%) | 16 (13.01%) |  |
|  | NA | 3 (1.64%) | 6 (4.88%) |  |
| CMV serostatus | Negative | 99 (54.10%) | 38 (30.89%) | **0.0001^2^** |
|  | Positive | 84 (45.90%) | 85 (69.11%) |  |
| EBV serostatus | Negative | 10 (5.46%) | 3 (2.44%) | 0.2549^4^ |
|  | Positive | 173 (94.54%) | 120 (97.56%) |  |
| Residual diuresis (ml/24h) | <500mL | 65 (35.52%) | 47 (38.21%) | 0.6721^2^ |
|  | >500mL | 103 (56.28%) | 65 (52.85%) |  |
|  | NA | 15 (8.20%) | 11 (8.94%) |  |
| DSA at day 0 | No | 167 (91.26%) | 102 (82.93%) | **0.0442^2^** |
|  | Yes | 16 (8.74%) | 21 (17.07%) |  |
| **Donor** | | | | |
| Sex | Female | 71 (38.80%) | 52 (42.28%) | 0.6375^2^ |
|  | Male | 110 (60.11%) | 70 (56.91%) |  |
|  | NA | 2 (1.09%) | 1 (0.81%) |  |
| Age (years) | | 52.99 (12.44) | 57.44 (13.05) | **0.0060^1^** |
| Living donor | No | 167 (91.26%) | 115 (93.50%) | 0.6189^2^ |
|  | Yes | 16 (8.74%) | 8 (6.50%) |  |
| **Transplantation** | | | | |
| Cold ischemia time (min) |  | 653.12 (341.16) | 751.59 (328.56) | **0.0074^1^** |
| ATG induction | No | 142 (77.60%) | 87 (70.73%) | 0.2216^2^ |
|  | Yes | 41 (22.40%) | 36 (29.27%) |  |
| Post-transplant creatinine nadir (µmol/L) |  | 228.40 (180.86) | 273.34 (192.44) | **0.0398^1^** |
| Tacrolimus / Cyclosporine | None | 1 (0.55%) | 0 (0%) | 0.0514^4^ |
|  | Ciclosporine | 7 (3.83%) | 12 (9.76%) |  |
|  | Tacrolimus | 175 (95.63%) | 111 (90.24%) |  |
| Maintenance with MMF | No | 116 (63.39%) | 67 (54.47%) | 0.1496^2^ |
|  | Yes | 67 (36.61%) | 56 (45.53%) |  |
| Maintenance corticosteroids | No | 4 (2.19%) | 4 (3.25%) | 0.7183^4^ |
|  | Yes | 179 (97.81%) | 119 (96.75%) |  |
| Quantitative data as mean (SD); qualitative data as n (%)  ¹ Wilcoxon rank-sum test; ² Chi-squared test; ³ Student’s t-test; ⁴ Fisher’s exact test | | | | |
